# Adipose-derived stem cell therapies for complex anal fistula: a systematic review and meta-analysis of randomized controlled trials

**DOI:** 10.3389/fmed.2025.1627065

**Published:** 2025-08-26

**Authors:** Meng Zou, Mengyao Xue, Yingjie Liu, Shijun Xia, Yongjin Chen, Zhaoyu Peng, Wenjiang Wu

**Affiliations:** ^1^Shenzhen Hospital (Futian) of Guangzhou University of Chinese Medicine, Shenzhen, China; ^2^The Sixth Clinical Medical School, Guangzhou University of Chinese Medicine, Shenzhen, China

**Keywords:** perianal fistula, adipose-derived mesenchymal stem cell, randomized controlled trial, adipose stem cells, systematic review and meta-analysis

## Abstract

**Purpose:**

This study aimed to systematically investigate the efficacy of adipose-derived stem cells (ASCs) in complex anal fistula treatment.

**Methods:**

This study systematically searched randomized controlled studies on the efficacy of ASCs in treating complex anal fistula published before June 2024 in PubMed, EMBASE, Web of Science, and Cochrane Library. Further, relevant journals were manually searched for relevant references, and two researchers independently performed literature search and screening, data extraction, and bias assessment. Stata version 12.0 was utilized to statistically analyze the healing rate of the anal fistula and the incidence of adverse events.

**Results:**

This study involved 1,056 patients from five randomized controlled trials, including 561 patients in the treatment group (ASCs/ASCs + fibrin glue) and 495 patients in the control group (fibrin glue/placebo). Meta-analysis revealed better short- and long-term efficacy of adipose mesenchymal stem cell treatment for complex perianal fistulas than in conventional treatment. However, no statistical difference was observed between mid-term and ultra-long-term treatments. Subgroup meta-analysis demonstrated a difference in efficacy between various cell doses. Further, all treatments with different sources of ASCs were superior to conventional quality.

**Conclusion:**

This study confirms that Adipose-derived Stem Cells can effectively improve short-term and long-term (1-year) clinical outcomes in patients with complex perianal fistulas, supporting their potential as a novel therapeutic strategy.

**Systematic review registration:**

https://www.crd.york.ac.uk/PROSPERO/view/CRD42024602327.

## 1 Introduction

Anal fistula is a complex etiology, treatment difficulty, and easy-to-recur perianal granulomatous inflammatory disease, complex anal fistula, including fistula through the external anal sphincter of >30% of the fistula (high sphincter between the sphincter and sphincter on the sphincter and outside the sphincter), the female anterior side of the anus fistula, as well as recurrent fistula or pre-existing fecal incontinence of the fistula, inflammatory bowel disease or radiation-induced fistula, etc. Clinically, the traditional treatment of surgical intervention is invasive, with slow recovery, and high postoperative fecal incontinence rates (1–3). The use of new minimally invasive treatment modalities that increase the cure rate of perianal fistulas, decrease the recurrence rate, and minimize the damage to the anus is an important issue that requires resolution in colorectal surgery.

Adipose-derived stem cells (ASCs) are a type of mesenchymal stem cells with self-renewal and multidirectional differentiation potential obtained by isolation and extraction from adipose tissues, which exhibit tissue regeneration and repair, inflammatory response inhibition, and immunomodulation functions (4, 5).

Stem cell transplantation has become a research hotspot in treating complex perianal fistulas, with the advantages of being minimally invasive, no anal sphincter injury, less pain, and shorter hospitalization time, in recent years (6). ASCs are from a wide range of sources, easy to obtain, highly plastic, and less immunogenic compared with other sources of MSCs, including bone marrow and umbilical cord (7). However, clinical studies on the use of ASCs for treating perianal fistulas in China and abroad are limited, and some systematic views and meta-analyses are supporting the treatment of complex perianal fistulas with MSCs, but the therapeutic role of ASCs has not been targeted. Therefore, this paper gathers relevant studies in recent years for meta-analysis to further investigate the therapeutic efficacy of ASCs in treating complex anal fistula for providing a basis for selecting further clinical treatment options in the future.

## 2 Information and methodology

### 2.1 Protocol and guidance

This study was conducted based on the Preferred Reporting Items for Systematic Reviews and Meta-Analyses (8) and Assessing the methodological quality of systematic reviews (9) guidelines. The protocol for this review has been registered with PROSPERO (CRD42024602327).

### 2.2 Search strategy

Randomized controlled trials (RCTs) of adipose-derived mesenchymal stem cells for treating complex anal fistulae from four databases, including Pubmed, Embase, Web of Science, and Cochrane Library, were counted from the time of their construction until June 2024, with language restriction to English. Search terms, including “perianal fistula,” “anal fistula,” “anorectal fistula,” “rectal fistula,” “intestinal fistula,” “adipose-derived mesenchymal stromal cells,” “stem cell,” “mesenchymal,” “randomized controlled trials,” and other synonymous search terms, were cross-searched in the above databases. Additionally, the literature included in existing similar systematic evaluations was browsed for additions to prevent omissions.

To distinguish this study from existing systematic reviews (10), our analysis exclusively focuses on RCTs evaluating adipose-derived stem cells (ASCs) rather than heterogeneous mesenchymal stem cell sources. Furthermore, we incorporated stringent PICOS criteria requiring MRI-confirmed fistula healing as a unified endpoint, which enhances comparability across studies—a methodological refinement not consistently applied in prior meta-analyses.

### 2.3 Inclusion and exclusion criteria

Inclusion criteria following the PICOS principles of evidence-based medicine were (1) study population (patient, P): ages of ≥18 years and a complex perianal fistula (either of the cryptoglandular origin or associated with Crohn’s disease) with a visible external opening (the disease activity index, the course of diseases, or the number of fstulas were not required); (2) intervention (intervention, I): adipose mesenchymal stem cell treatment or adipose mesenchymal stem cell combined with fibrin glue treatment; (3) comparison intervention (C): placebo treatment or fibrin glue treatment; (4) outcome indicator (O): cure rate; (5) study design (S): a randomized controlled trial.

Exclusion criteria were (1) non-RCT studies; (2) case series reports, non-original studies, reviews or commentaries, and related studies with lacking outcome data; (3) inclusion of study populations from the same cohort of subjects from the same research organization; (4) duplicate publications, with only one selected for inclusion.

### 2.4 Information extraction

A cross-section of two researchers independently conducted literature screening and data extraction. A senior researcher was involved in the discussion and gave judgment on the results in case of disagreement or disagreement during the checking process. Data extraction included authors, year of publication, diagnostic information of patients, sample size of the trial and control groups, interventions, and anal fistula healing at different time points. Data extraction for each eligible study were title, authors, year of publication, grouping method, blinding, sample sizes of trial and control groups, intervention, ASC dose, and anal fistula healing at different time points. The primary endpoint of pain was anal fistula healing at different time points. Fistula healing was evaluated by investigating digital photographs of the external fistula opening and clinical examination reports. Healing was predefined as the absence of drainage through the external openings (whether occurring spontaneously or under externally applied pressure) and complete re-epithelialization of the external openings.

### 2.5 Quality assessment

The Risk of Bias (RoB2) risk assessment tool (11) provided by the Cochrane Collaboration was utilized to evaluate the risk of bias in the included studies. The assessment included randomization method, allocation concealment, blinding, outcome variable completeness, selective study result reporting, and other biases. Each evaluation item was evaluated as “yes,” “no,” or “unclear.” Each of the above evaluation items was judged as low risk, possible risk of bias, and high risk based on the performance of the included literature.

### 2.6 Statistical analysis

Stata version 12.0 software was applied to conduct a meta-analysis of healing in patients with postoperative anal fistula at different time intervals to calculate the relative risk (RR) and the associated 95% confidence intervals (CIs) and to compare the efficacy between the intervention and control groups. A random-effects model was selected for meta-analysis. *P*-values of <0.05 indicated statistically significant differences.

## 3 Results

### 3.1 Literature search results and basic features

**Figure 1:**
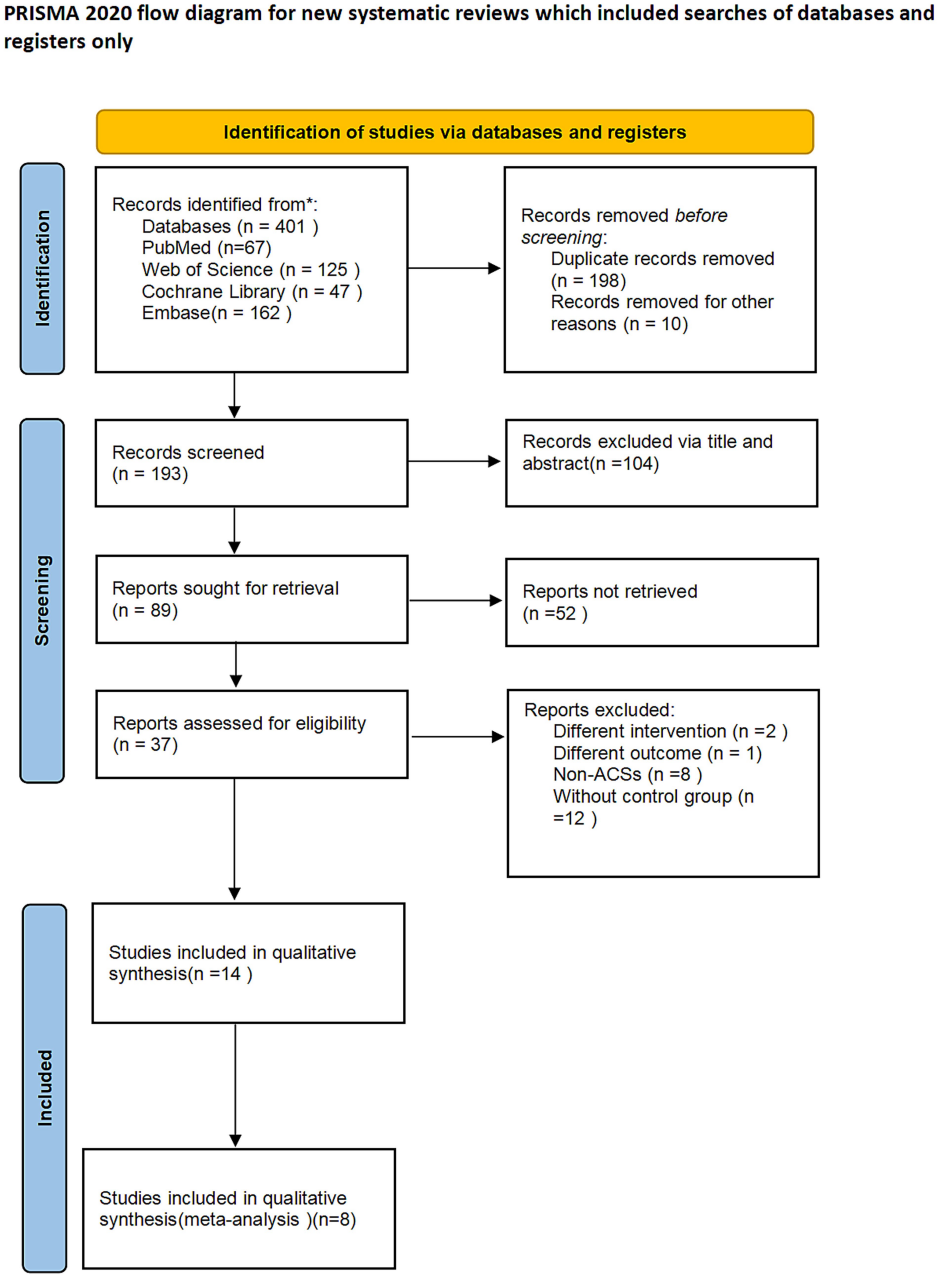
Flow diagram of the study selection.

Literature screening process. A total of 401 pieces of literature were initially inspected, duplicates were deleted based on the title and abstract, and case reports, experimental studies (including animal experiments, cellular experiments, etc.), reviews, systematic evaluation, and meta-analysis measures that do not match the number of research literature were excluded. The full text of the screening was read, non-RCs were then deleted, and articles that did not meet the inclusion and exclusion criteria were excluded. Finally, 380 articles were excluded and 8 articles were included in this review. These eight articles were based on five clinical trials (results from the same clinical trial were combined in the same group) (Figure 1).

The follow-up period of the five studies ranged from 8 weeks to 2 years, yielding cure rates at various stages, which can be utilized to analyze the efficacy of adipose mesenchymal stem cell therapy. The treatment and control groups consisted of 561 and 495 cases, respectively, resulting in a total number of 1,056 cases included in the statistical analysis. The applied ASC dose in the trials ranged from 2 × 10^7^ to 20 × 10^7^. In three studies [Garcia-Olmo et al. (12), Herreros et al. (13), Garcia-Arranz et al. (14)], participants who were defned as unhealed after the frsttime treatment would receive the second time treatment. Unhealed participants would receive a double dose of the frst-time intervention in the second treatment in two studies [Garcia-Olmo et al. (12), Herreros et al. (13)]. The same dose of intervention would be used to treat in the second treatment in the study [Garcia-Arranz et al. (14)].

At follow-up, persistent drainage from the external fistula opening or failure to achieve complete re-epithelialization (despite reductions in septic symptoms and improvements in local perianal appearance) were considered treatment failures. The difference in general information in all patients between the treatment and control groups was not statistically significant (all *p* > 0.05), and the baseline characteristics were comparable. Endpoint definitions for fistula healing varied across studies: Garcia-Olmo et al. (12) and Garcia-Arranz et al. (14) defined healing as the absence of drainage through the external openings (whether occurring spontaneously or under externally applied pressure) and complete reepithelialization of external openings. Herreros et al. (13) defined healing as the absence of drainage through the external openings, complete re-epithelization of external openings, and the absence of collections >2 cm by MRI. Panés et al. (15–19) and Serclova et al. (20) defined healing as closure of all treated external openings (Clinical assessment of closure was defined as the absence of draining despite gentle finger compression.) and the absence of collections larger than 2 cm of the treated perianal fistulas in at least two of three dimensions, confirmed by masked central MRI. Table 1 shows the baseline information and clinical characteristics of the eligible studies.

**Table 1:** Baseline information and clinical characteristics of the included literature.

						Intervention				
References	Publication date	Randomization and blinding	Type of perianal fstulas	Groups (interventionl group vs. control group)	N (interventionl group)	Experimental group (fixed cell dose)	Control group	ASC source	MRI (outcome assessment)	Follow-up Phase (post-treatment)
Garcia-Olmo et al. (12)	2009	1:1; open-label	(1). Cryptoglandular (2). Crohn’s disease	I: ASCs + fibrin glue C:fibrin glue	49 (24 vs. 25)	First: 2 × 107 + fbrin glue Second: double dose	First: fbrin glue Second: double dose	Autologous	No	8 weeks* 1 year
Herreros et al. (13)	2012	1:1:1; single-blind	Cryptoglandular	I a: ASCs I b: ASCs+ fibrin glue C: saline solution	183 (64, 60 vs. 59)	First: 2 × 107 , 2 × 107 + fbrin glue Second: double dose	First: fbrin glue Second: double dose	Autologous	Yes	12 weeks 24/26 weeks*1 year
Panés et al. (15–19)	2016; 2017; 2019; 2020; 2022	1:1; double-blind	Crohn’s disease	I: ASCs C: placebo	212 (107 vs. 105)	12 × 107	Saline solution: 24 mL	Allogenic	Yes	24 weeks* 1 year 2 years
Garcia-Arranz et al. (14)	2020	1:1; single-blind	Cryptoglandular	I: ASCs+ fibrin glue C: fibrin glue	45 (23 vs. 21)	First: 10 × 107 + fbrin glue Second: 10 × 107 + fbrin glue	First: Fibrin glue (2–5 mL) Second: Fibrin glue (2–5 mL)	Autologous	No	16 weeks* 52 weeks 2 years
Serclova et al. (20)	2024	1:1; double-blind	Crohn’s disease	I: ASCs C: saline solution	568 (283 vs. 285)	12 × 107	Saline solution: 24 mL	Allogenic	Yes	24 weeks* 1 year 2 years

*Endpoint indicator.

### 3.2 Quality assessment of the literature

**Figure 2:**
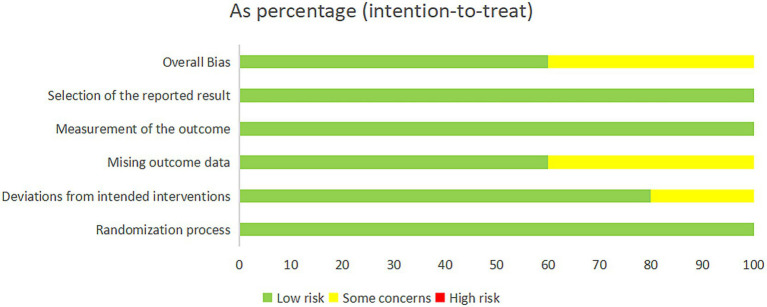
Risk of bias summary.

**Figure 3:**
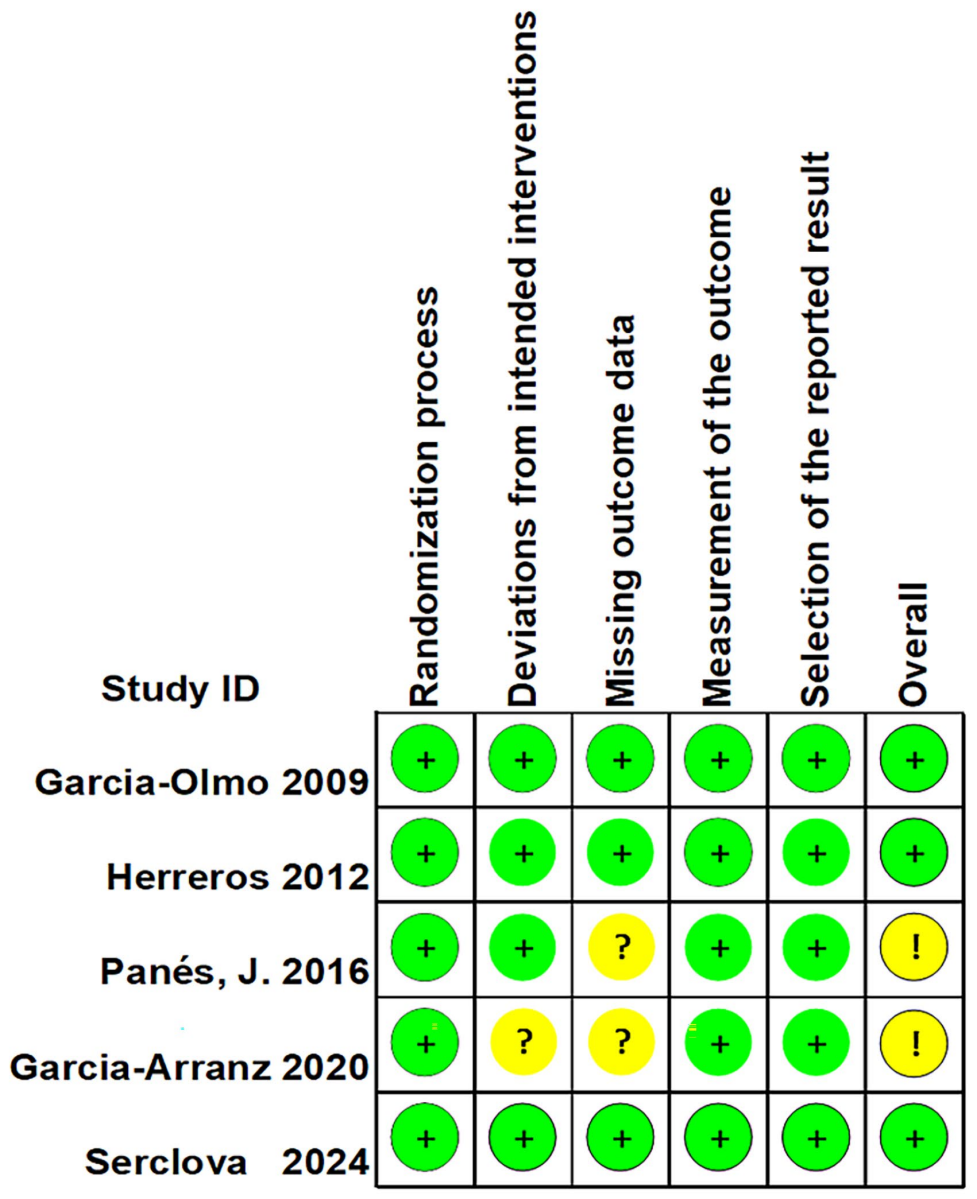
Risk of bias graph.

The included studies were evaluated with the RoB2 risk assessment tool and were overall at moderate risk (Figures 2, 3).

### 3.3 Efficacy of ASCs in treating complex perianal fistulas

The primary study endpoint in this study is the healing rate. Fistula healing is the absence of spillage of incoming fluid through the external opening (either spontaneously or under externally applied pressure) and complete re-epithelialization of the external opening.

### 3.4 Meta-analysis of the postoperative anal fistula healing rate

**Figure 4:**
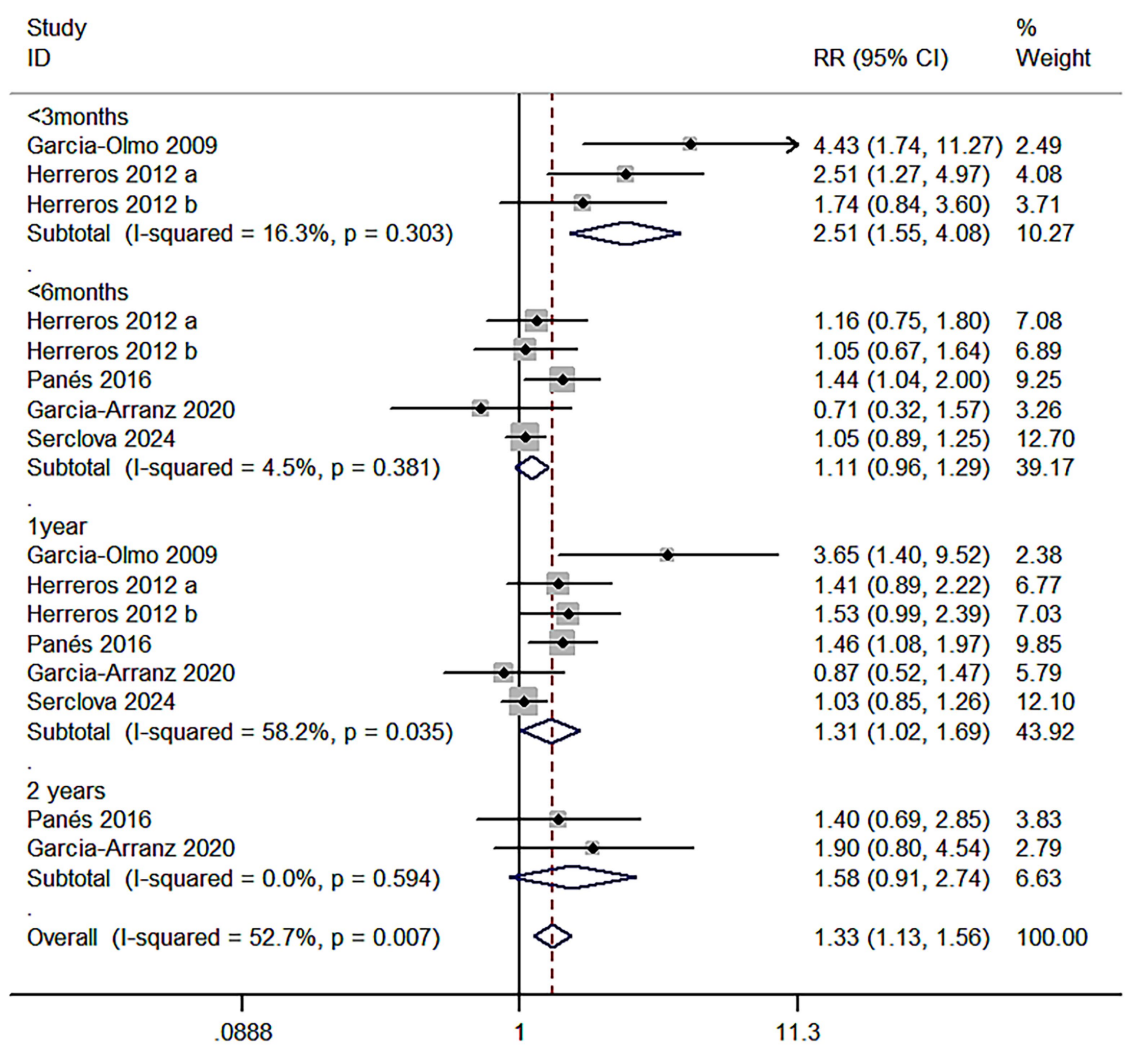
Forest plot comparing the healing rate of the anal fistula at different postoperative time points in the two groups of patients.

Meta 1: Short-term follow-up of the efficacy of ASCs on perianal fistula (≤3 months) intervention group (ASCs/ASCs + fibrin glue): control group (fibrin glue) ratio was 148:143. Z = 3.74 and *p* < 0.000 indicated that adipose mesenchymal stromal cells are superior to conventional treatment for anal fistula (R = 2.51; 95% CI: 1.55–4.08). I^2^ test (I^2^ = 16.3%, *p* = 0.303) revealed low heterogeneity (Figure 4).

Meta 2: Mid-term follow-up of the efficacy of ASCs on perianal fistula (<6 months post-treatment) the adipose MSCs group (ASCs/ASCs + fibrin glue):control group (fibrin glue or placebo) ratio was 537:529. Z = 1.45 and *p* = 0.147 revealed that the efficacy of adipose mesenchymal stromal cells on anal fistulae was not statistically different from fibrin glue or saline treatment (R = 1.11; 95% CI: 0.96–1.29). I^2^ test (I^2^ = 4.5%, *p* = 0.381) revealed low heterogeneity (Figure 4).

Meta 3: Long-term follow-up of the efficacy of ASCs in perianal fistulas (approximately 1 year post-treatment). The adipose mesenchymal stem cell group (ASCs/ASCs + fibrin glue): control group (fibrin glue or placebo) ratio was 516:532. Z = 2.09 and *p* = 0.036 demonstrated that ASCs were more effective than conventional treatment for anal fistula (R = 1.31; 95% CI: 1.02–1.69). I^2^ test (I^2^ = 58.2%, *p* = 0.035) revealed high heterogeneity (Figure 4).

Meta 4: Ultra-long-term follow-up phase (>2 years post-treatment) for the efficacy of ASCs in perianal fistula, with two eligible studies. The adipose MSC group (ASCs/ASCs + fibrin glue): control group (fibrin glue or placebo) ratio was 45:34. Z = 1.63 and *p* = 0.103 showed that the efficacy of ASCs on anal fistula was not statistically different from conventional treatment (R = 1.58; 95% CI: 0.91–2.74). I^2^ test (I^2^ = 0.0%) revealed low heterogeneity (Figure 4).

**Figure 5:**
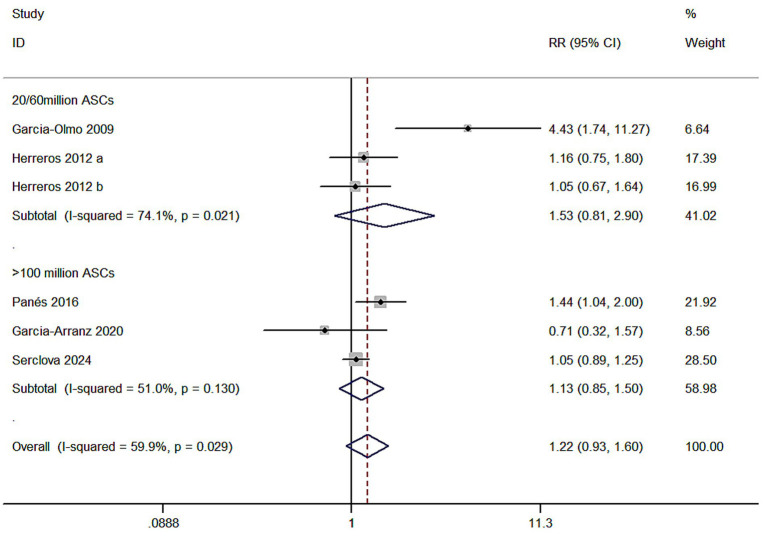
Forest plot of the efficacy of different adipose mesenchymal stem cell doses.

Meta 5: Different ASC doses for complex anal fistulae. In two of the five studies, the ASCs treated were low dose (20/60 × 10^7^), with the intervention group (ASCs/ASCs + fibrin glue): control group (fibrin glue) ratio of 148:143. Three items were high dose (120 × 10^7^, 100/200 × 10^7^, and 120 × 10^7^). The intervention group (ASCs/ASCs + fibrin glue): control group (fibrin glue) ratio was 413:411. The results revealed that the low-dose ASCs subgroup (RR = 1.53, 95% CI: 0.81–2.90; Z = 1.30, *p* = 0.193) and the high-dose ASCs subgroup (RR = 1.13, 95% CI: 0.85–1.50; Z = 0.83, *p* = 0.404) exhibited no statistical difference in efficacy from the control group. Within-group heterogeneity between the two groups was significant (I^2^ = 74.1%; I^2^ = 51%) for the total events and the I^2^ test (I^2^ = 59.9 > 50%) revealed a high degree of heterogeneity, indicating that the different dosage study types are not a significant influencing factor in causing significant heterogeneity (Figure 5).

**Figure 6:**
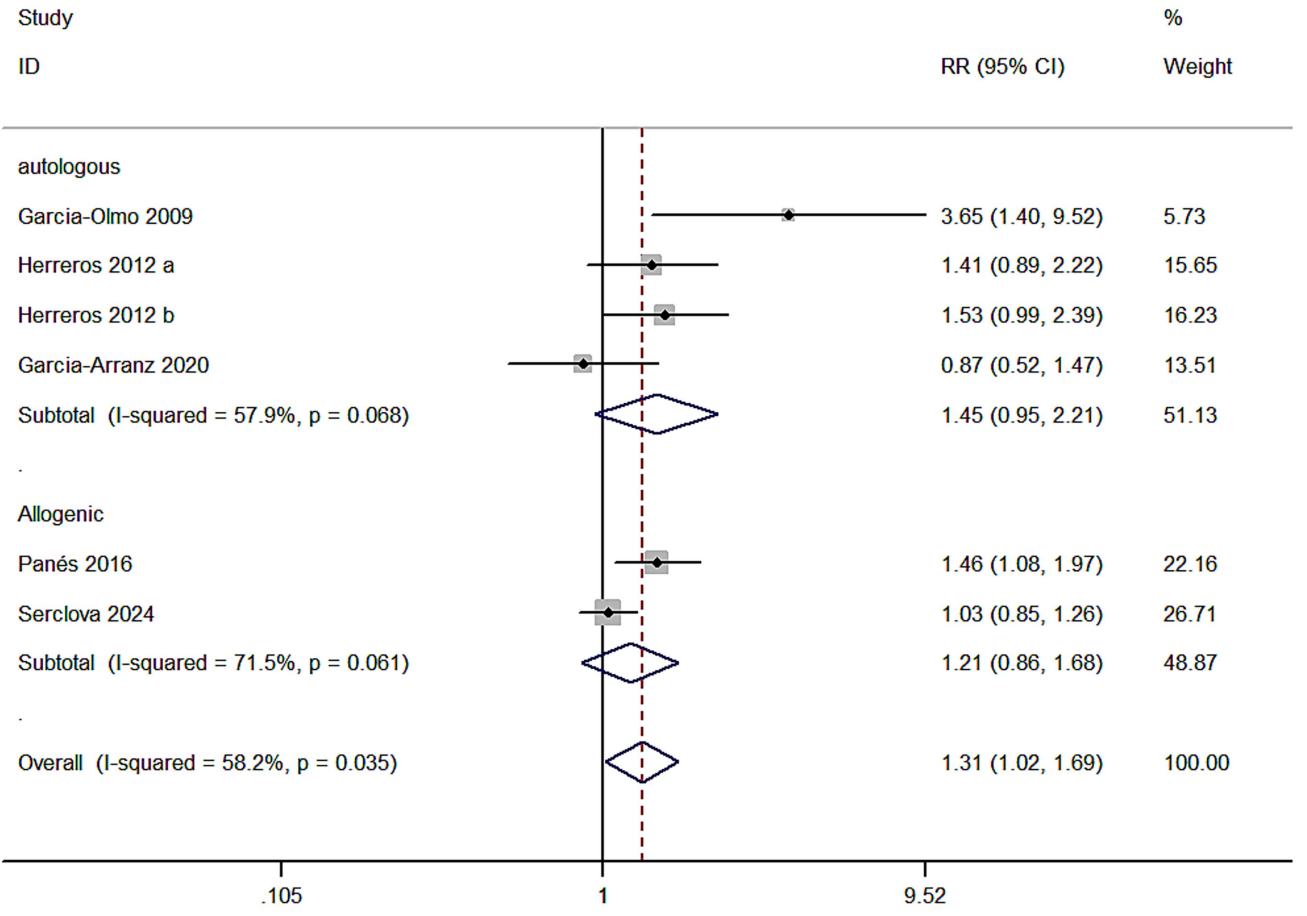
Forest plot of the efficacy of different sources of ASCs.

Meta 6: Different sources of ASCs for treating complex perianal fistulas. Three studies focused on autologous ASCs for treating complex perianal fistulas (Autologous). Heterogeneity was observed in the meta-analysis (I^2^ = 57.9% > 50%) in the autologous MSC subgroup (ASCs/ASCs + fibrin glue) and the control group (fibrin glue) with 128 and 146 individuals, respectively. Two studies investigated allogeneic MSCs for treating complex perianal fistulas, with 386 and 386 individuals in the allogeneic MSC subgroup and control group, respectively, and heterogeneity was present in the meta-analysis (I^2^ = 71.5% > 50%). A random-effects model was utilized. The healing rate was better in both subgroups than the control group (55.5% versus 40.0%; 45.1% versus 39.4%), but the autologous MSC subgroup (RR = 1.45, 95% CI: 0.95–2.21, Z = 1.70, *p* = 0.08) and the allogeneic MSC subgroup (RR = 1.21, 95% CI: 0.86–1.68, Z = 1.09, *p* = 0.275) demonstrated no statistically significant results. Overall analysis showed significant efficacy (*p* = 0.035), suggesting possible heterogeneity between the two groups. Further meta-regression was performed to test for differences between subgroups, which were significant (*p* = 0.032), and the efficacy of autologous adipose-derived stem cell therapy was superior to that of allogeneic adipose-derived stem cell therapy (RR values were lower in the allogeneic group). Grouping variables explained 30% of the heterogeneity (R^2^ = 30.0%), and the remaining heterogeneity remained high (I^2^ = 45.3%) (Figure 6).

**Figure 7:**
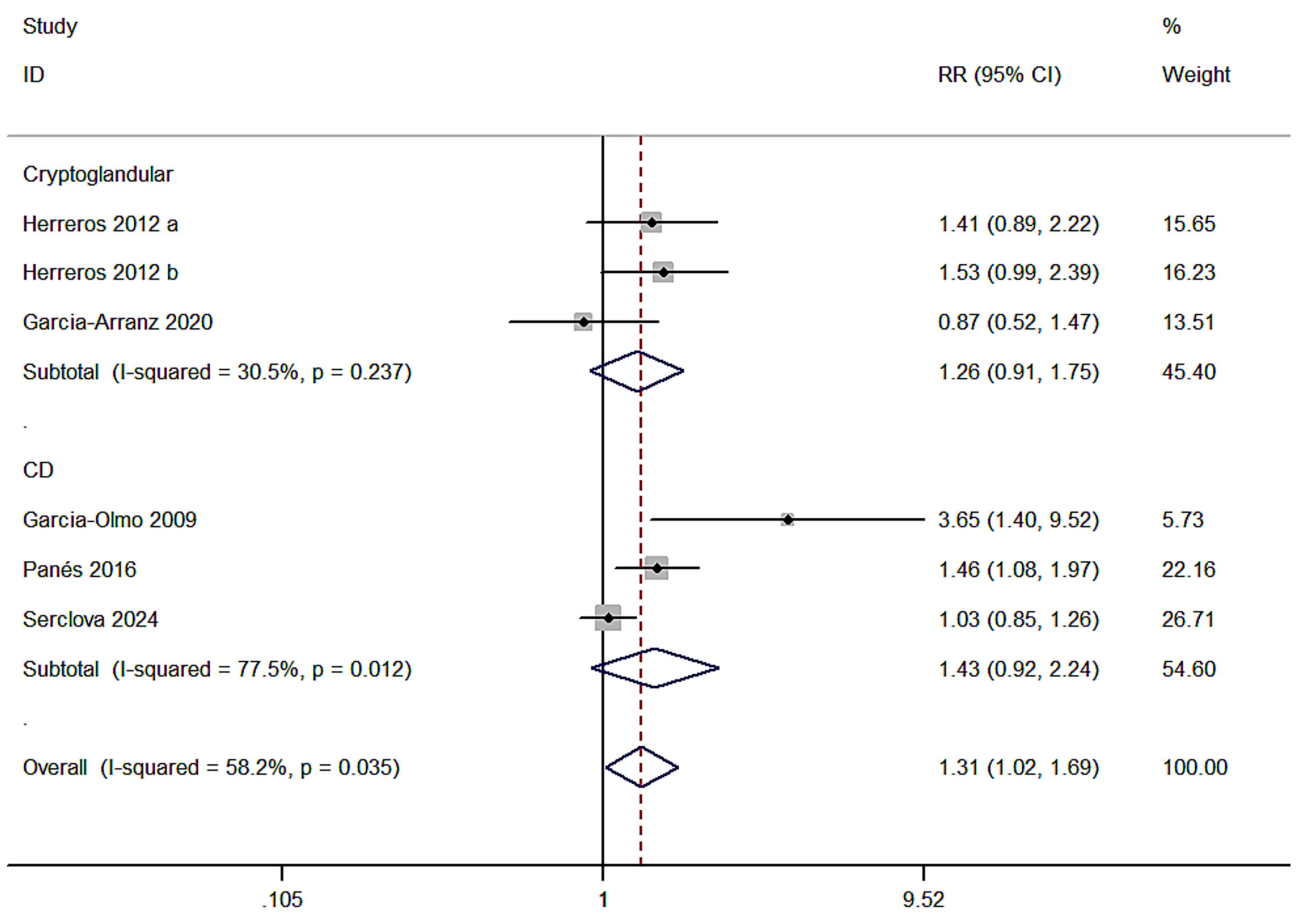
Forest plot of the efficacy of different types of complex perianal fistulas.

Meta 7: ASCs for diferent types of complex perianal fstulas (CD or cryptoglandular). Two of five studies reported ASCs for the treatment of cryptoglandular infection fstulas. There were 104 versus 121 participants in the ASCs group (ASCs/ASCs + fibrin glue) and control group (fbrin glue/saline solution). Three of five studies reported ASCs for the treatment of CD fstulas. There were 410 versus 411 participants in the ASCs group and control group. A random model was applied. In cryptoglandular subgroup, RR = 1.26, 95% CI (0.91, 1.75) and Z = 1.41, *p* = 0.160 indicated no statistical signifcance of this subgroup even though the HR of MSCs group was superior compared to the control group (54.81% versus 41.32%). In CD group, the HR of MSCs group also indicated no statistical signifcance (RR = 1.43, 95% CI: 0.92, 2.24; Z = 1.59, *p* = 0.113; 45.83% versus 37.96%) (Figure 7).

### 3.5 Evidence quality evaluation

We used the GRADE pro system to evaluate the quality of evidence for the primary outcomes: short, intermediate, long-term, and ultra-long-term healing rates. In the GRADE quality of evidence assessment, RCTs were preset to the highest level of evidence and processed according to five downgrading factors. The results suggested that the quality of evidence for short, intermediate, long-term, and ultra-long-term cure rates was at a moderate level, whereby the final evidence for the study was of moderate quality. See Table 2.

**Table 2:** Evaluation of GRADE in complex anal fistula with ASCs therapy.

ACSs compared to placebo for complex and fistula Patient or population: patient with complex anal fistula Settings: Intervention: ACSs Comparison: Placebo						
Outcomes	Illustrative comparative risks* (95%CI)		Relative effect (95%)	No. of Participants (Studies)	Quality of evidence (GRADE)	Comments
	Assumed risk Placebo	Corresponding risk ACSs				
Healing rate-<3 months	Study population		RR 2.51 (1.55 to 4.08)	300 (2 studies)	⨁⨁⨁⊖ moderate	
	154 per 1000	386 per 1000 (238 to 628)				
	Moderate					
	153 per 1000	384 per 1000 (237 to 624)				
Healing rate-<6 months	Study population		RR 1.11 (0.96 to 1.29)	1066 (4 studies)	⨁⨁⨁⊖ moderate	
	418 per 1000	464 per 1000 (401 to 539)				
	Moderate					
	373 per 1000	414 per 1000 (358 to 481)				
Healing rate-<1 year	Study population		RR 2.51 (1.02 to 1.69)	537 (5 studies)	⨁⨁⨁⊖ moderate	
	387 per 1000	507 per 1000 (395 to 654)				
	Moderate					
	379 per 1000	496 per 1000 (387 to 641)				
Healing rate-<2 years	Study population		RR 2.51 (0.91 to 2.74)	79 (2 studies)	⨁⨁⨁⊖ moderate	
	324 per 1000	511 per 1000 (294 to 886)				
	Moderate					
	332 per 1000	525 per 1000 (302 to 910)				

*The basis of assumed risk (e.g. the median control group risk across studies) is provided in footnotes. The corresponding risk (and its 95% confidence interval) is based on the assumed risk in the comparison group and the relative effect of the intervention (and its 95%CI).

CI, Confidence interval, RR, risk ratio.

GRADE working group grades of evidence.

High quality: Further research is very unlikely to change our confidence in the estimate of effect.

Moderate quality: Further research is likely have an impact on our confidence in the estimate of effect and may change the estimate.

Low quality: Further research is very likely to have an important impact on our confidence in the estimate of effect and is likely change the estimate.

Very Low quality: We are very uncertain above the estimate.

### 3.6 Publication bias

As the number of included studies was < 10, we could not evaluate publication bias.

## 4 Discussion

ASC is a type of MSC with self-renewal and multi-directional differentiation potential isolated from adipose tissue, which exhibits a large reserve in the human body and is easy to obtain. It can self-regenerate and multi-directionally differentiate as well as promote the secretion of many angiogenic factors and anti-apoptotic factors (21).

Japanese and European scholars have used ASCs, with the emergence of ASCs, to construct engineered adipose tissue for soft tissue defect repair, breast augmentation, facial fillers, and breast reconstruction, with good results (22). Subsequently, ASCs have been used in various systemic diseases related to tissue repair and reconstruction, autoimmunity, and humoral immunity.

ASC transplantation for treating anal fistula exhibits wound healing that involves inflammation, epithelial formation, neovascularization, proliferation, and collagen matrix formation (23, 24). Its mechanism of action is mainly categorized into the following aspects: (1) Multidirectional Differentiation Potential: ASCs are pluripotent stem cells with robust proliferative capacity. ASCs differentiate into adipocytes, chondrocytes, osteoblasts, myoblasts, neuroglial cells, and neuronal cells under different inducing conditions (25, 26), providing a cellular foundation for tissue regeneration. (2) Paracrine/Autocrine Secretory Functions: ASCs secrete various cytokines, such as fibroblast growth factor 2 (FGF-2), vascular endothelial growth factor (VEGF), epidermal growth factor (EGF), etc., in an autocrine or paracrine manner (27). Hypoxic microenvironments further amplify cytokine secretion, accelerating tissue repair processes. (3) Immunomodulatory Effects: Wound healing is based on the inflammatory response, but an excessive inflammatory response can graft local tissue healing or even induce healing arrest or tissue necrosis (28). ASCs restore immune homeostasis by: Downregulating pro-inflammatory mediators (e.g., TNF-*α*, IL-6) while upregulating anti-inflammatory factors (e.g., TGF-*β*, IL-10, prostaglandin E2) (29–35); ASCs interact with various immune cells in the immune system and affect their differentiation and activation (31, 32). (4) Angiogenesis and Tissue Remodeling: ASCs drive neovascularization via VEGF-dependent pathways, re-establishing local blood supply to hypoxic regions. Concurrently, they activate fibroblasts to enhance granulation tissue formation and collagen matrix deposition (23, 24, 27), collectively addressing the pathophysiological barriers in refractory wound healing.

These mechanisms highlight ASCs’ multifaceted role in bridging cellular plasticity with microenvironmental regulation, offering a rationale for their application in complex perianal fistula management.

In 2003, Garcíaolmo et al. (36) first tested the tissue around the rectovaginal fistula in perianal disease, and the wound healed after the injection treatment. In 2005, Garcíaolmo et al. (37) evaluated the safety and feasibility of ASCs for treating Crohn’s disease combined with anal fistula, and eight patients with Crohn’s disease combined with complex anal fistula were treated and six of them had complete closure of the fistula, and the other two patients exhibited improvement of localized septic symptoms without adverse effects after 8 weeks. The fistula was completely closed in six patients, and localized pus symptoms improved without adverse effects in two patients. Panés et al. (6) revealed that 50% of the patients with Crohn’s disease combined with anal fistula treated with expanded allogeneic adipose-derived mesenchymal stem cell transplantation demonstrated significant lesion improvement after 24 weeks, compared to 34% in the placebo group (*p* < 0.05). The lesions improved significantly in terms of adverse effects in the stem cell treatment group compared to the placebo group (*p* < 0.05). Further, the patients in the placebo group exhibited no adverse effects, and the stem cell treatment group demonstrated a significant improvement. Adverse effects indicated a significant reduction in the stem cell treatment group compared to the placebo group (17% vs. 29%). Herreros et al. (15) localized use of autologous expanded fat-derived stem cell grafts in combination with lipoprotein gels causing a fistula healing rate of 40% in complex perianal fistulas at 6 months postoperatively, and 50% at 1 year postoperatively, with no adverse effects. Until now, several systematic reviews and meta-analyses have been published on perianal fistulae treatment with stem cells, including both RCT and single clinical trials, whereas this study included only RCTs on the treatment of complex anal fistulae with ASCs to improve accuracy.

In this study, the effectiveness of ASC treatment at different follow-up stages was initially differentiated through a meta-analysis to evaluate whether adipose MSC treatment has short- and long-term benefits. Only complex fistulas were required to be detected in the study participants to expand the sample size, and disease activity index and disease duration were not evaluated. As for intervention, significant heterogeneities existed in the studies concerning the cell dosage, diferent sources of ASCs and aetiologies, so we performed subgroup analyses appropriately to fgure out whether those factors would influence treatment efficacy.

The results of Meta 1 (R = 2.51; 95% CI: 1.55–4.08; z = 3.74, *p* < 0.000) and Meta3 (R = 1.31; 95% CI: 1.02–1.69; z = 2.09, *p* = 0.036) indicated that the treatment was effective in the patients’ short- and long-term follow-up periods (38.5% versus 15.4%; 47.5% versus 38.7%). Meta 2 (R = 1.11; 95% CI: 0.96–1.29; Z = 1.45, *p* = 0.147) and Meta 4 (R = 1.58; 95% CI: 0.91,–2.74; Z = 1.68, *p* = 0.103) results regarding the mid-term follow-up and the ultra-long-term follow-up period indicated no significant difference in the treatment measures between the two group (R = 1.11; 95% CI: 0.96–1.29). However, in terms of total events, the cure rate was significantly higher in both ASC groups compared to the control group (46.4% vs. 41.8 and 53.3% vs. 32.4%, respectively) and participant attrition may increase with prolonged follow-up periods, and data collected during extended follow-up phases may become increasingly susceptible to bias and reduced reliability. Figures 2, 3 display this condition the long-term and over-long-term follow-up results in the studies by Garcia-Olmo (12) were defined with attrition bias. Therefore, we deemed that ASCs therapy was more efective than traditional therapy in the medium-term follow-up phase.

We compared the outcomes across five studies: In Studies of Garcia-Olmo (12), Herreros et al. (13), Panés (15–19), and Serclova (20), the cure rates in the ASCs group remained consistently higher than those in the control group over time. Study by Garcia-Arranz et al. (14), however, the cure rates in the ASCs group were lower than the control group at both 16 weeks and 52 weeks (fortunately, without statistically significant differences). Notably, during the 2-year follow-up phase, the ASCs group demonstrated superior efficacy, with a significant reduction in long-term recurrence cases directly associated with fibrin glue. The investigators hypothesized that these contrary results might be linked to the “cleaning surgery (deep curettage)” protocol and the “elimination of placebo effects through blinded outcome assessment by surgeons.” However, in earlier trials, blinded assessments were similarly implemented, yet their results did not exhibit such contradictions as observed in Study of Garcia-Arranz et al. (14). Consequently, Garcia-Arranz et al. (14) concluded that ASCs therapy for anal fistulas is safe and may enhance long-term sustained healing of perianal fistulas.

Subgroup analyses were performed based on the extracted data, focusing on cell dosage, different sources of ASCs, cell types, and aetiological characteristics. To maximize data inclusion, the hazard ratio (HR) at the 1-year follow-up was selected as the uniform metric in Meta 6 and 7, whereas HR comparisons in Meta 5 were conducted according to the prespecified outcome assessment timepoints of individual studies.

Meta 5 results revealed that the differences between the low-dose ASCs subgroup, the high-dose subgroup and the control group were not significant (RR = 1.53, 95% CI: 0.81–2.90; RR = 1.13, 95% CI: 0.85–1.50). However, the cure rate of the ASCs group was better than that of the control group in terms of the total events. Therefore, we conclude that ASCs therapy can be used as a complementary treatment to conventional therapy, and the efficacy of different doses of MSCs may vary, but the optimal cell dose for anal fistula treatment remains unknown. Garcia-Arranz et al. (14), in 2020, concluded no dose–response relationship. Meanwhile, the other investigators argued a best-suited dosage for treatment and that a larger number of cells could behave immunogenic, thereby increasing clearance or deactivation of the cells (38–40).

The sources of ASCs are an arguing point, which concerning safety problems. Meta 6 results indicated no statistically significant difference between autologous ASCs and allogeneic ASCs for complex anal fistulas treatment and the control. The cure rate of both groups was better than the control group in terms of total events. Our meta-analysis demonstrated that ASC therapy from all sources exhibited superior efficacy compared to conventional treatments for complex anal fistulas, although conclusive evidence regarding variations in effectiveness among different ASC sources remains elusive. Meta-regression analysis revealed a statistically significant difference between subgroups (*p* = 0.032), with autologous ASCs demonstrating greater therapeutic benefits than allogeneic ASCs. However, these findings should be interpreted with caution due to limitations inherent in the subgroup analyses, including small sample sizes, insufficient statistical power to adjust for potential confounders (e.g., ASC dosage variations), and heterogeneity in study protocols. Neoplastic transformation remains a critical safety concern in stem cell-based therapies, particularly following evidence implicating stem cells in tumorigenesis and cancer progression (41). However, to date, no neoplastic events associated with ASCs therapy have been documented in clinical studies.

Meta 7 results indicated ASCs therapy was ineffective for CD fistulas (RR = 1.43, 95% CI: 0.92, 2.24; Z = 1.59, *p* = 0.113; 45.83% versus 37.96%) and cryptoglandular (RR = 1.26, 95% CI: 0.91, 1.75; Z = 1.41, *p* = 0.160; 54.81% versus 41.32%). Few studies included were the limitation of our study, though this subgroup analysis could not offer enough evidence. We still tend to maintain that ASCs therapy was effective for cryptoglandular fstulas.

Moreover, adverse events (AEs) and serious adverse events (SAEs) are critical indicators for evaluating treatment safety. Although a systematic pooled analysis of all AEs/SAEs was not performed, a comprehensive review of the full-text articles confirmed the association of these events with stem cell therapy. Heterogeneity in the definitions of AEs/SAEs across studies was noted. In the studies of Garcia-Olmo et al. (12), Herreros et al. (13), and Panés et al. (12–16) explicitly differentiated between treatment-related AEs and non-treatment-related AEs. Importantly, no AEs/SAEs were directly linked to ASCs therapy, and no mortality was reported during follow-up across all studies.

AEs were predominantly mild to moderate in severity, with common manifestations including proctalgia, abscesses, and perianal infections, which were primarily attributed to the underlying disease progression or surgical procedures rather than the stem cell therapy itself. In the study of Garcia-Olmo et al. (12), among four SAEs, only one case of perianal abscess was associated with fibrin glue, while none were linked to ASCs. The Study of Herreros et al. (13) reported four SAEs related to the study procedures (e.g., liposuction), none of which were attributable to the therapeutic intervention. In Study of Panés et al. (12–16), both the intervention and control groups exhibited five cases of severe treatment-related anal abscesses (5% each) at the 24-week follow-up. During the extended follow-up period (up to 104 weeks), four new SAEs (intervention: 3; control: 1) were reported, all associated with fistulas or abscesses. The study of Serclova et al. (17) further corroborated the safety profile of DVS, demonstrating consistency with previous data and no emerging safety concerns.

The strengths of the systematic evaluation of this study mainly involved the following two points. (1) The articles included are high-quality RCTs, and the results have a high degree of confidence. (2) The literature search and screening were conducted, discussed, and decided under the standardized multiple authors’ crossover, which maximally prevents selective bias, and therefore the results are more reliable. The limitations are as follows. (1) Factors that may bias the results include the small number of included literature, the different disease types and duration, and the various treatment assessment systems of each clinical center. (2) The routine surgical methods of anal fistula varied in each study. (3) The included RCTs did not report sufficient economic indicators; thus, the associated cost-effectiveness could not be systematically assessed. (4)The current evidence on long-term outcomes remains limited, necessitating the inclusion of more robust data from large-scale multicenter trials to validate these findings.

ASC transplantation for complex anal fistulae exhibits the advantages of being less invasive, not damaging the anal sphincter, reusable, and having a higher cure rate than other biologics. Current Challenges in Stem Cell Therapy for Complex Anal Fistula:

At present, the existing research on adipose-derived stem cells (ASCs) for complex anal fistula is hampered by significant heterogeneity in study protocols, including inconsistencies in cell sources, dosage standards, disease classifications, and efficacy evaluation methods. These discrepancies limit the comparability of findings and impede clinical translation.

Key issues include: (1) Dosage Variability: Administered ASC doses vary substantially across studies (e.g., 20 million to 120 million cells), with a lack of systematic investigations into dose–response relationships. (3) Unclear Treatment Frequency and Administration Routes: The comparative efficacy of single versus repeated treatments remains undefined, and evidence supporting the selection of delivery methods (e.g., direct injection vs. Bio-material carriers) is insufficient. (4) Limited Long-Term Efficacy Data: Most studies focus on outcomes within 2 years post-treatment, while high rates of loss to follow-up compromise the availability of robust long-term data. (5) Incomplete Safety Profiling: Critical endpoints, such as long-term anal functional outcomes (e.g., fecal continence), are inadequately reported. (6) Special Subgroup Analyses: Data on high-risk populations, including rectovaginal fistula patients and pregnant individuals with Crohn’s disease-related fistulas, are notably absent, restricting ASC applicability in these cohorts.

Strategies for Future Optimization: (1) Dose Standardization: Conduct phase I/II dose-escalation trials to establish optimal ASC dosage ranges and validate dose-dependent therapeutic effects. (2) Dynamic Treatment Protocols: Implement adaptive regimens guided by real-time monitoring of fistula healing (e.g., MRI or ultrasound), with supplementary injections administered if closure is incomplete. Compare bio-material carriers (e.g., hydrogels vs. scaffolds) for their effects on cell retention and survival. (3) Enhanced Long-Term Follow-Up: Design multi-center prospective cohort studies with predefined follow-up intervals (3/6/12/18/24/36 months) and extend observation periods to ≥5 years. Incorporate genomic stability assessments to evaluate tumorigenic risks. (4) Special Subgroup Focus: for rectovaginal fistulas, integrate 3D MRI reconstruction to quantify closure depth in phase II trials. Establish international pregnancy registries to track maternal-fetal outcomes (e.g., preterm birth rates, congenital anomalies) in Crohn’s disease patients receiving ASC therapy. (5) Standardized Data Reporting: Adopt core outcome measures, including: Primary Efficacy Endpoint-complete fistula closure rate, mandating MRI confirmation. Patient-Reported Outcomes-Validated instruments such as the Anal Fistula Quality of Life Scale (AF-QoL).

In summary, ASCs promote fistula wound healing and improve the healing rate of complex anal fistula through mechanisms, such as inducing differentiation, regulating inflammatory immunity, promoting neovascularization, and activating fibroblasts, which were further investigated and promoted in the clinic.

## 5 Conclusion

This meta-analysis establishes ASCs as a clinically viable strategy for complex perianal fistulas, demonstrating significant short-term (≤3 month) and sustained 1-year efficacy—particularly with autologous cells. However, unresolved challenges include: ultra-long-term (>2 years) durability uncertainty due to attrition bias and dose standardization and delivery optimization.

## Data Availability

The original contributions presented in the study are included in the article/supplementary material, further inquiries can be directed to the corresponding author.
